# Stainless Steel in Municipal Sewage—How to Recognize Favorable Corrosion Conditions

**DOI:** 10.3390/ma16206637

**Published:** 2023-10-11

**Authors:** Paweł Lochyński, Magdalena Domańska, Robert Dziedzic, Kamila Hamal

**Affiliations:** 1 Institute of Environmental Engineering, Wroclaw University of Environmental and Life Sciences, pl. Grunwaldzki 24, 50-363 Wroclaw, Poland; pawel.lochynski@upwr.edu.pl (P.L.); kamila.hamal@upwr.edu.pl (K.H.); 2 Faculty of Mechanical Engineering, Wroclaw University of Science and Technology, Lukasiewicza 5, 50-371 Wroclaw, Poland; robert.dziedzic@pwr.edu.pl

**Keywords:** austenite, biological corrosion, chromium–nickel steel, delta ferrite, fluorescence in situ hybridization (FISH), pitting corrosion

## Abstract

While chromium–nickel steel is known to be extremely resistant to corrosion, the occurrence of certain factors can unfortunately initiate an uncontrolled corrosion process. This paper presents samples made of 304 stainless steel containing delta ferrite that have been exposed to wastewater for 18 months. Samples placed above the surface of the wastewater (A-series) were intensively corroded. Samples half-submerged in the wastewater and periodically fully submerged at higher effluent flows through the screenings and grit separator (B-series) only suffered minor mechanical erosion. No significant changes in the tested surface were observed on samples fully submerged in wastewater (C-series). The results indicated that the observed pitting corrosion of samples placed above the surface of the wastewater was a consequence of the presence of bacteria in a wet hydrogen sulfide environment. The fluorescence in situ hybridization method showed that either the sludge taken from the wastewater, or from the surface of samples submerged in wastewater exhibited increased amounts of bacteria from the δ-proteobacteria class, indicating the presence of microorganisms involved in the reduction of sulfur or sulfate compounds. A new approach to microbiological evaluation by determining classes of bacteria may be a promising tool for evaluating wastewater in terms of aggressiveness and recognizing favorable corrosive conditions.

## 1. Introduction

The problem of material destruction due to corrosion, which occurs under the influence of chemical and electrochemical reactions, concerns primarily metals [1], but also concretes and plastics [2]. The improvement of steel composition was supposed to contribute to a reduction in this phenomenon which entails hefty economic losses, estimated at 4% of the GNP of developed countries [1]. However, due to their chemical composition and phase composition, stainless steels (SS) are characterized by different levels of corrosion resistance. Chromium–nickel steel is considered to be exceptionally resistant to corrosion, but the presence of phases or inclusions other than austenite in the structure could lower the corrosion resistance [3]. Usually, 304 steel is made of 100% austenite, but sometimes, it can be a mix of austenite and a small amount of ferrite. This is not the ferrite found in carbon steel, but a high-temperature form known as delta (δ) ferrite [4]. The use of the label “delta” ferrite implies that this is an equilibrium body-centered cubic (bcc) phase that forms during solidification, analogous to the delta phase of pure iron that exists from a melting point of 1532 °C down to 1493 °C. Delta ferrite, by contrast, is a thermodynamically stable phase that exists in equilibrium with austenite, and as such, has a different composition to the austenite [5]. Ferrite reduces the steel’s tendency to form solidification cracks during cooling. It is not uncommon for 304 castings (CF8) to contain 8% to 20% ferrite. The amount of delta ferrite in hot, deformed 304 steel can be adjusted by changing the chemical composition (e.g., chromium-to-nickel equivalent) or by changing the temperature and strain of deformation [6]. A small amount of delta ferrite in SS can be very beneficial, causing the steel to become immune to hot cracking [7] and to exhibit improved resistance to the formation of sulfur-containing liquid films. The reasons for this include the fact that ferrite can dissolve more sulfur and phosphorus than austenite, meaning that they are retained in the solution rather than being available to form liquid films along the grain boundaries. The presence of quite a small amount of ferrite increases the grain boundary area, so that any liquid films must spread over a greater area and can no longer form a continuous liquid film. The 100% austenitic steels do not have this advantage [4]. Although Rhouma et al. [8] did not record any major impact of delta ferrite on corrosion at low contents (1%) of delta ferrite, increased delta ferrite affected the shortened fatigue life of 304 L steel [9]. A study by Mohammed-Ali et al. [10] showed that 304 L steel, which contained 4 to 6 wt.% delta ferrite, was significantly affected by atmospheric pitting corrosion under MgCl_2_ droplets. Tests on 304 L steel containing delta ferrite in the structure showed that, in an environment containing 3% HCl + 8.6 g NaCl, the largest corrosion pits appeared precisely where delta ferrite was present [3]. The corrosion resistance of austenitic steels containing delta ferrite varies greatly, as it depends not only on the amount of ferrite in the alloy, but also on the chemical composition of the alloy and the type of corrosion medium. There are also several additional factors that can alter the corrosion resistance of ferrite-containing stainless steels, e.g., phosphorus and sulfur impurities, chromium-depleted zones, and a lower concentration of Cr in the austenite phase [10]. Similarly, based on STEM/EDS analysis, the ferrite phase of super duplex stainless steel was characterized by higher Cr, Mo and W contents compared with austenite [11].

Bacterial biofilms form on technical devices. This is a phenomenon commonly encountered in both natural environments and industrial processes. Heterogeneous biofilm and associated bacteria form complex biological systems that can cause chemical changes at the metal/biofilm interface, e.g., the formation of gradients in pH, dissolved oxygen, chlorides and sulfates. Compared with drinking water, sewage quickly stimulates the development of microorganisms on SS surfaces. Microorganisms adhere to the surface, which is the pre-corrosion step of microbiologically influenced corrosion (MIC), and then multiply and finally form a biofilm on the surface. Biofilm is particularly resistant to removal and influences the kinetics of the corrosion process [12]. Despite the stimulation of bacterial growth by sewage, the number of microorganisms deposited on pipes immersed in fresh water increases as the immersion time increases [13]. Ziadi et al. [14] carried out research on the composition of biofilm immersed in raw sewage and cleaned 304 L stainless steel pipes. In both media, an aerobic biofilm was initially formed in which the bacteria *Caldimonas*, *Caulobacter*, *Terriglobus* and *Edaphobacter* (iron-oxidizing bacteria) and the genera *Bacillus*, *Sulfurimonas*, *Synthroobacter* and *Desulfobacter* (sulfur-oxidizing bacteria—SOB) were present. After the formation of large amounts of EPS, which hindered the access of oxygen, anaerobic bacteria such as *Desulfovirga, Desulfovibrio, Desulfuromusa, Desulfococcus* and *Desulfosarcina* (sulfate-reducing bacteria—SRB) began to grow. One of the most harmful microorganisms is *Acidithiobacillus ferrooxidans*, the presence of which accelerates corrosion by three to six times in abiotic acidic environments [15]; an example of such an environment is the pit solutions in 304, where the pH is very low (pH < 2) [16].

While in the literature, we often find laboratory tests describing corrosion of steel containing only austenite, few test results exist for steel containing other components such as delta ferrite or carbides. In addition, there is also a lack of analyses of operating facilities, where process control is difficult. The aim of this study was to evaluate the phenomenon of corrosion of 304 steel containing delta ferrite exposed to real sewage and a humid environment containing hydrogen sulfide, as well as the influence of other factors such as microorganisms present in the medium in contact with the steel. The novelty of this study is the suggestion of an approach to microbiological evaluation by identifying classes of bacteria as a tool for evaluating wastewater for aggressiveness. This idea was intended to recognize favorable corrosive conditions. The tests were carried out in a facility where fast-progressing corrosion of the screenings and grit separator was previously observed [17].

## 2. Materials and Methods

### 2.1. Characteristics of the Test Facility

The screenings and grit separator in which the study was conducted, and the prevailing conditions, are described in detail in [17]. Previous studies have evaluated the corrosion damage of a stainless steel screen separator. The technical facility appeared to the authors to be a suitable experimental set-up for conducting tests on stainless steel samples containing delta ferrite. Moreover, these issues have not been fully clarified in the literature.

The screenings and grit separator combine the function of a spiral screen and horizontal sand trap with a grit separator. Domestic wastewater flows into the treatment plant via a pressurized sewer system. In the first stage, they are directed to the screen expansion chamber, where screenings are separated, mechanically compressed and stored in waste containers. Wastewater from the screen flows into the grit chamber, where sedimentation of sand and other easily sedimentable suspended solids takes place. The sand and suspended solids are dewatered and mechanically fed into the container. The average effluent flow through the grit screen during the experiment was between 160 and 190 m^3^/day. The maximum throughput is 1000 m^3^/day. Selected physico-chemical parameters of the effluent flowing into the screenings and grit separator from the end of the experiment included pH > 7, chlorides > 500 mg/dm^3^, sulphates above 60 and periodically above 250 mg/dm^3^, phosphates > 20 mg/dm^3^, total suspended solids > 1200 mg/dm^3^, alkalinity > 19 mval/dm^3^ and total acidity > 2 mval/dm^3^, while no mineral acidity was detected [17]. There is gravity ventilation and mechanical exhaust ventilation in the screen room.

### 2.2. Description of the Experiment

**Figure 1:**
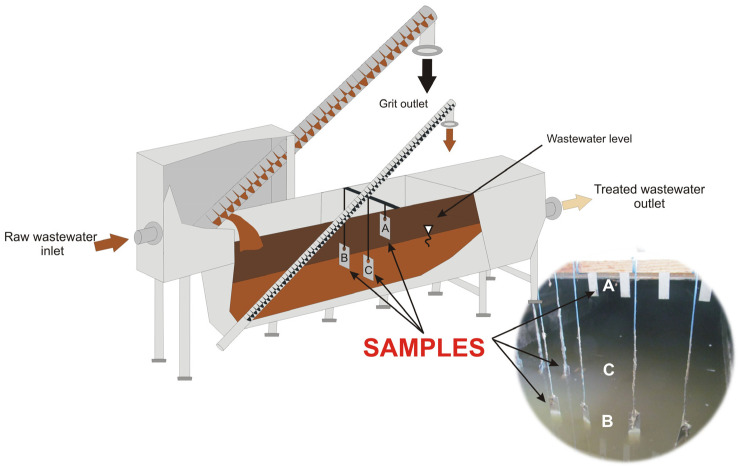
Simplified diagram of the location of samples for exposure tests in zones A, B and C of the screenings and grit separator chamber and an actual picture from the installation. Arrows indicate the position of stainless steel test specimens from a particular series.

Twelve AISI 304 stainless steel test specimens, four in each zone (A, B and C), were suspended from polyethylene ropes attached to the frame of the screenings and grit separator below the protective covers (Figure 1). Samples from the A-series were not in direct contact with the wastewater. The B-series samples were half-submerged in wastewater and periodically fully submerged at higher effluent flows through the screenings and grit separator. The C-series samples were submerged in wastewater. After 18 months of exposure, the samples were pulled out and the sediment accumulated on the surface was subjected to microbiological and microscopic (EDX) analysis. After having been cleaned, the samples were subjected to microscopic examination, and weight loss was determined.

### 2.3. Material Characterization

The tests were carried out on samples made of cold-rolled AISI 304 stainless steel with a thickness of 1.5 mm. Optical surface profiler imaging was conducted using the Sensofar S Neox that allows for obtaining quantitative information about the surface roughness. Surface average roughness (Sa) values were within a range of 0.18–0.22 µm. The chemical composition of the samples used in the study is specified according to the certificate obtained from the stainless steel manufacturer (Table 1).

**Figure 2:**
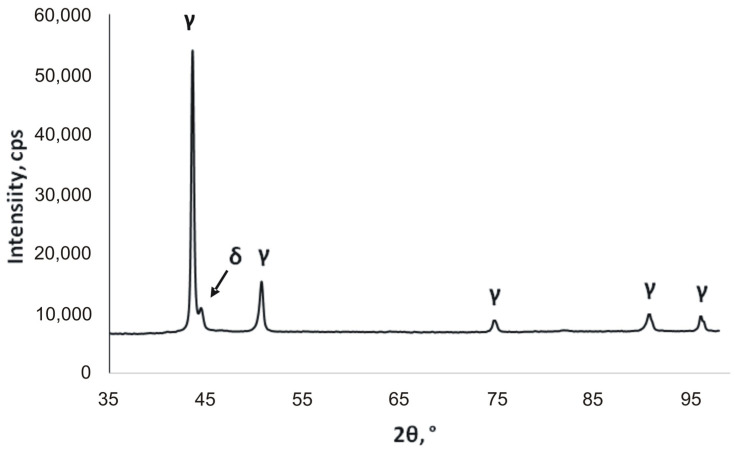
Characteristics of the material used for the study. X-ray diffraction pattern of stainless steel 304 sample (δ—ferrite, γ—austenite).

The size of the specimens to be tested was 30 mm wide and 90 mm long, with a hole of a 12 mm diameter located 5 mm away from the upper edge. The analysis of the microstructure and corrosion pits formed was carried out using an Olympus OLS4000 confocal microscope (Olympus Corporation, Tokyo, Japan). Phase analysis was carried out on a Rigaku Miniflex 600 X-ray diffractometer (Rigaku, Tokyo, Japan) equipped with a copper lamp and HyPix strip detector. In order to check the presence of the individual phases in the samples, XRD analysis was carried out on a metallographic deposit made on the cross-section of the sample, along the rolling direction. For an angle of 2Θ equaling 44.44°, a typical A2 lattice peak with an intensity of 11,001 cps appeared. The conducted measurements showed that delta ferrite was present in the tested samples in addition to the austenite phase (Figure 2). The presence of delta ferrite in the structure is the result of incomplete transformation into austenite during the fabrication process.

### 2.4. Bacteria Identification

Wastewater and sludge that formed on the surface of the C-series samples, hanging in a chamber of the grit separator for 18 months, were analyzed. The sediment was stained with Live/DEAD reagent to be tested for the presence of living and dead microorganisms using the Thermo Fisher’s Live/DEAD reagent according to the manufacturer’s protocol. The sediment was then fixed with 4% paraformaldehyde and then with FISH before quantitative analyses were performed. The FISH procedure began with a 1:20 dilution of the sample, while the standard protocol according to Amann et al. [18] was used in the study. DAPI reagent, general probe EUB338 and specific probes were used for the study, as shown in Table 2. The hybridization process consisted of incubating the cells at 46 °C in a solution of NaCl, Tris/HCl, SDS (sodium dodecyl sulfate) and formamide. The incubation time was 24 h. Washing was performed in a water bath at 48 °C. In the study, 4 classes of Proteobacteria were identified (Table 2) in order to indicate which classes predominated in the sludge and the wastewater. Probes ALF968 and DELTA495a, identifying α- and δ-Proteobacteria, respectively, were labeled with 6-carboxyfluorescein dye (6-FAM), while probes Gam42a and BET42a, identifying β- and γ-Proteobacteria, respectively, were labeled with carboxy-X-rodamine (ROX). For the EUB338 probe, different dyes were used depending on the need to visualize the distribution of each class of bacteria against all bacteria.

For the quantitative analysis, 15 images, each of sediment stained with general probe, specific probe and DAPI reagent, were taken sequentially. Analyses were performed for 4 specific probes twice. A total of 360 images were taken. A Nikon Eclipse Ni-E C2 confocal microscope and NIS-Elements AR 4.30 software were used for the analysis. The appropriate threshold (the level of fluorescence corresponding to the presence of the probe’s signal) was then defined for each probe. In this procedure, binary images were obtained for which fluorescent areas were automatically counted. After determining the area of fluorescence, the percentage ratio of a specific probe area to DAPI was calculated [19,20]. In this way, 15 different percentage ratios were obtained, for which medians and percentiles of 25–75% were defined in Statistica 13.3. The analysis of wastewater was performed in the same way.

## 3. Results

### 3.1. SEM Analysis

**Figure 3:**
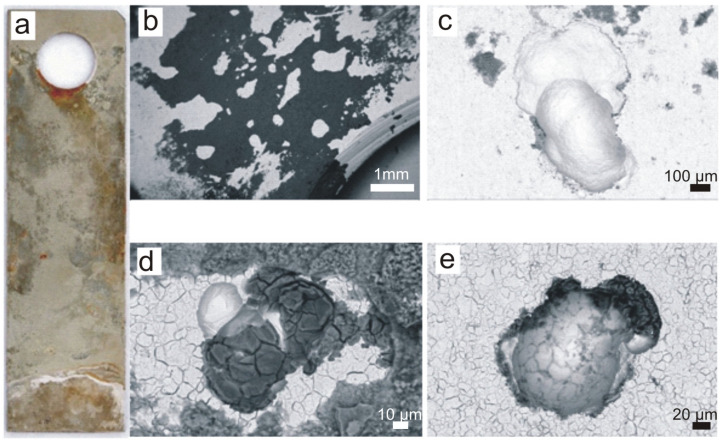
A-series sample after 18 months of exposure: (**a**) macroscopic image, (**b**) deposit near the sample suspension hole, (**c**) largest pitting found on the surface of the sample measuring 615 × 736 µm, (**d**) pitting with deposit, (**e**) pitting without deposit. Scale bars: 1 mm (**b**), 100 μm (**c**), 10 μm (**d**), 20 μm (**e**).

The surface of the A-series sample after eighteen months of exposure inside the screenings and grit separator chamber in the wastewater treatment plant is shown in Figure 3. Figure 3a shows the entire surface, where large areas of corrosion product coverage are visible. Figure 3b shows the area near the sample suspension hole with a large amount of dark sediment visible. Figure 3c shows the largest pitting found on the surface with dimensions of 615 × 736 µm and a maximum depth of approximately 370 µm. Figure 3d shows a pitting ‘covered’ by sediment and another pitting with a small amount of sediment (Figure 3e).

**Figure 4:**
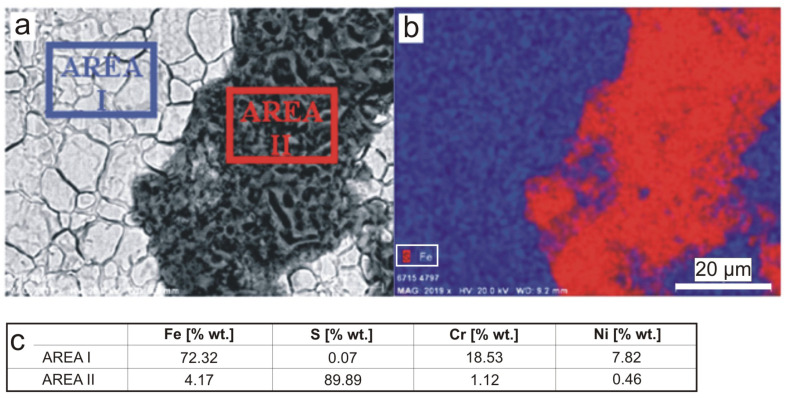
Analysis of the chemical composition of the deposit from the A-series sample after 18-month exposure: (**a**) area analyzed with areas of quantitative analysis marked, (**b**) distribution of iron (blue) and sulfur (red) in the area analyzed, (**c**) quantitative results of the main elements from the areas analyzed. Scale bar 20 μm.

The results of the surface chemical composition analysis presented in Figure 4 show that the dark areas are dominated by sulfur, whose content in the examined area (AREA II in Figure 4a and red area in Figure 4b) was determined to be 89.89% by weight. Analysis of the chemical composition of the substrate material showed the typical content of the main alloying elements for 304 steel, i.e., chromium (18.53 wt.%) and nickel (7.82 wt.%) (Figure 4c).

**Figure 5:**
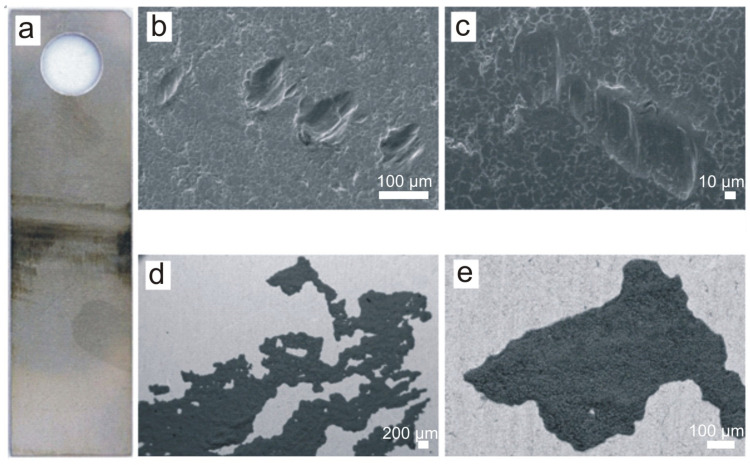
B-series sample after 18 months of exposure: (**a**) macroscopic image, (**b**,**c**) microscopic images with minor surface defects of a mechanical damage nature, (**d**,**e**) deposit. Scale bars: 100 μm (**b**), 10 μm (**c**), 200 μm (**d**), 100 μm (**e**).

**Figure 6:**
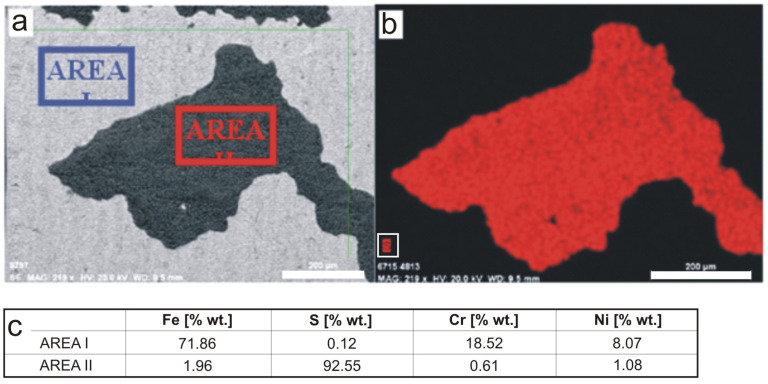
Analysis of the chemical composition of the deposit from the B-series sample after 18-month exposure: (**a**) area analyzed with areas of quantitative analysis marked, (**b**) distribution of sulfur (red) in the area analyzed, (**c**) quantitative results of the main elements from the areas analyzed. Scale bar 200 μm.

Images of the surface of the B-series sample after eighteen months of exposure in the screenings and grit separator suggest that for the most part, there were no obvious signs of pitting corrosion on the surface of the sample (Figure 5a). The few surface defects (shown in Figure 5b,c) were, similar to the C-series, of a mechanical nature. Through SEM analysis, it was found that the dark area visible in the central part of the sample (Figure 5d,e) is a deposit whose main component is sulfur. A detailed analysis is shown in Figure 6.

As in the case of the deposits on the A-series sample, the chemical composition analysis showed that the dark areas were dominated by sulfur, whose content in the examined area (AREA II in Figure 6a and red area in Figure 6b) was determined to be 92.55% by weight. Analysis of the chemical composition of the substrate material showed the typical contents of the main alloying elements for 304 steel, i.e., chromium (18.52 wt.%) and nickel (8.07 wt.%) (Figure 6c).

**Figure 7:**
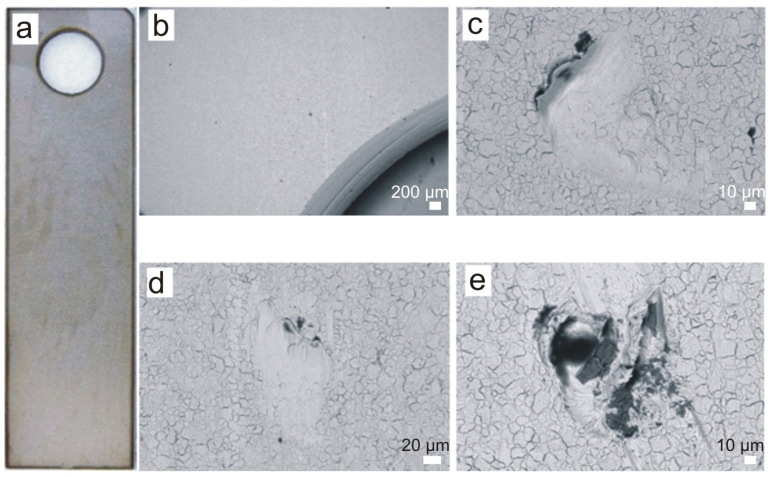
C-series sample after 18 months of exposure: (**a**) macroscopic image, (**b**) area near the sample hanging hole, (**c**,**d**) areas with small surface defects of a mechanical damage nature, (**e**) defect with a small amount of sediment. Scale bars: 200 μm (**b**), 10 μm (**c**), 20 μm (**d**), 10 μm (**e**).

Images of the surface of the selected C-series sample after the exposure period are shown in Figure 7, with the surface shown having no obvious corrosion products (Figure 7a). Analysis via electron microscopy (Figure 7b–e) confirmed that there were no pitting corrosion effects on the surface, and that the small surface deformations present were of a minor mechanical nature. Samples immersed in the screenings and grit separator chamber were exposed to the impact of fine solids, e.g., sand, flowing together with the wastewater through the unit. Only a few areas (Figure 7e) showed the presence of a small amount of deposits. Despite the aggressive environment, 304 steel containing delta ferrite immersed in the wastewater solution did not corrode as quickly as the series of samples that were not immersed in the wastewater solution (series A). Osoba et al. [21] also showed that a higher delta ferrite content in 304 steel does not necessarily cause a decrease in corrosion resistance in a 1 M sulfuric acid environment, or in a 1 M sodium chloride environment.

### 3.2. Surface Roughness, Weight Loss of Samples and Pitting Geometry

The raw sample had a surface roughness ranging from 0.18 to 0.22 µm. After 18 months of exposure, the samples from series A, B and C in the “no defect” area were characterized by ranges of 0.21–0.36 µm; 0.21–0.32 µm; and 0.22–0.33 µm, respectively. In the area of “large” defects in the A -series, the parameter Sa was 0.54–6.48 µm, and for the samples in group B, as a result of erosion, Sa was 0.26–0.66 µm. The weight loss results of the tiles showed a significant difference between tiles immersed in the effluent compared with tiles suspended above the effluent table. On the tiles immersed in the wastewater, pitting was negligible, and the damage looked more like mechanical damage resulting from the impact of mineral particles on the surface of the tiles during the intensive flow of the wastewater. The results of corrosion changes on the A-series samples indicate that the corrosion phenomenon is difficult to control. The weight loss of one sample in this series was 40 mg, and the average for the other three was 5 mg. In comparison, the samples after 18 months of operation showed an average weight loss of 1.3 mg for series C and 2.4 mg for series B. In a humid environment containing H_2_S, after damage/destruction of the passive layer under favorable temperature and humidity conditions, insufficiently efficient ventilation and the influence of microorganisms, an intensification corrosion may occur from the corrosion focus propagating deep into the pits. The pitting depth for the raw sample before the 18-month exposure in the sieve-sander was 4–9 µm. For the A-series samples, the pitting depth increased by an average of 100–150 times after the 18-month exposure. SEM measurements and analyses were performed for 10 pits of each A-series sample. These samples were characterized by varying pitting depths ranging from 10 to 400 µm. This is due to the characteristic uneven pitting formation on the surface of the samples. The median of the 10 measurements for the test samples was 78; 99; 132; and 136 µm for a pitting depth of 20·10^3^; 24·10^3^; 26·10^3^; and 42·10^3^ µm, 2 for the pitting area and 0.5·10^6^; 1.5·10^6^; 1.8·10^6^; and 1.9·10^6^ µm^3^ for pitting volume, respectively.

### 3.3. Microbial Identification and Quantitative Assessment

**Figure 8:**
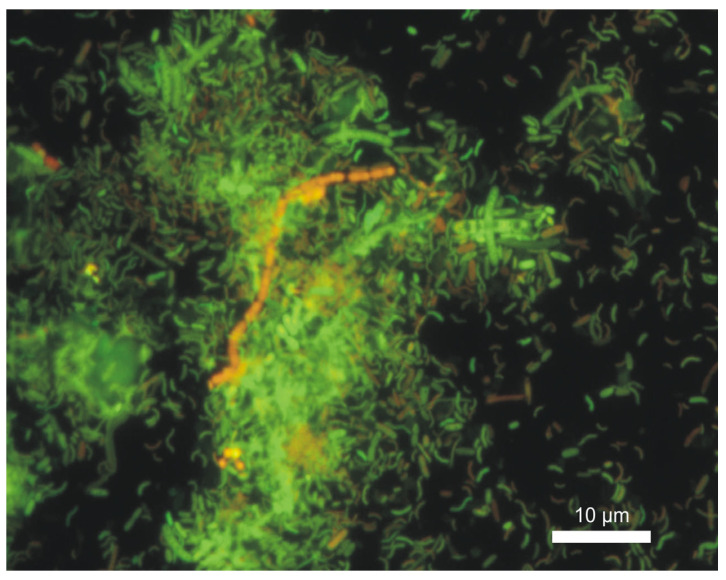
Live/DEAD staining of sediment from the surface of the B-series sample submerged in wastewater. Scale bar 10 µm.

Sulfate- and sulfur-reducing bacteria (SRB), as well as filamentous bacteria, are mainly responsible for the decomposition of sulfur-containing organic compounds, while the course of oxidation and reduction processes depends on the conditions that bacteria need for growth [2]. The Live/DEAD staining confirmed the presence of living bacteria, filamentous bacteria and protozoa in the sludge taken from the B- and C-series samples submerged in wastewater flowing into the grit separator. Figure 8 depicts most of the living bacteria from the B-series.

**Figure 9:**
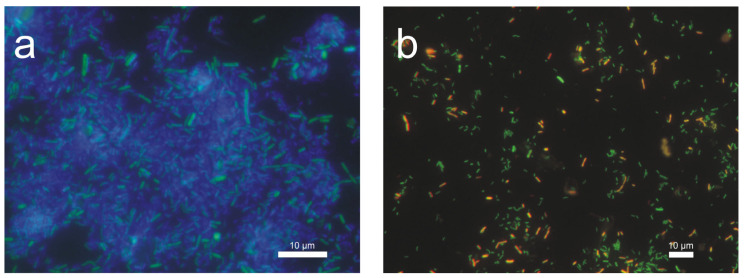
FISH hybridization of sediment taken from the surface of the C- (**a**) and B-series (**b**) sample submerged in wastewater using the specific probe DELTA495a (green) and GAM42a (red). Image of δ-Proteobacteria in the background of DAPI-stained bacteria (**a**) and δ-Proteobacteria and γ-Proteobacteria (**b**). Scale bar 10 μm.

In environmental samples, searching for specific bacterial species is very time-consuming. The use of next-generation sequencing (NGS) provides information on the full spectrum of microorganisms and confirms that the biofilms are mainly dominated by Deltaproteobacteria [22], but it is an expensive analysis. FISH is a useful technique for identifying SRB bacteria [23], and the identification of specific classes can indicate the presence of specific groups of bacteria responsible for creating conditions favorable for corrosion. FISH analysis showed the presence of all analyzed classes of Proteobacteria. Figure 9a shows the abundance of the δ-Proteobacteria class in the background of all bacteria stained with DAPI (blue). Figure 9b depicts δ-Proteobacteria and γ-Proteobacteria. Pictures were obtained by superimposing the images.

**Figure 10:**
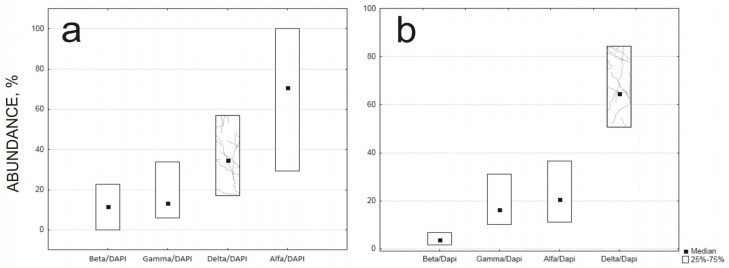
Quantitative analysis of each class of Proteobacteria (Alfa-, Beta-, Gamma- and Deltaproteobacteria) in the wastewater sample taken from the grit separator (**a**) and sediment taken from the surface of the C-sample submerged in wastewater for 18 months (**b**).

The study results showed that about 15% of the Gammaproteobacteria class were observed in the wastewater and sewage sludge relative to all bacteria stained with DAPI. The Gammaproteobacteria class includes sulfur-oxidizing bacteria, e.g., *Acidithiobacillus thiooxidans*. Results from the literature indicate that similar amounts of the class may be observed in the wastewater [24]. Deltaproteobacteria, which include SRB-reducing bacteria, were present at a level of 30% in the wastewater (Figure 10a) and dominated in the sewage sludge (Figure 10b). The results of the quantitative analysis showed that a significant group of microorganisms were bacteria from the δ-Proteobacteria class, confirming their important role in hydrogen sulfide production. The δ-Proteobacteria class is usually the least prominent in municipal wastewater compared with other classes of Proteobacteria [25].

## 4. Discussion

The weight loss results of the tiles showed a significant difference between samples submerged in wastewater compared with tiles suspended above the wastewater surface. On the plates immersed in the wastewater, pitting was negligible, while the damage looked more like mechanical damage resulting from mineral particles hitting the surface of the plates during the intensive wastewater flow. The results reflect the situation in the screenings and grit separator, where the greatest damage was observed above the surface of the wastewater [17]. However, in a study by Fischer et al. [26] on the effect of periodic immersion and total immersion of 304 L and 316 L steel fragments in ocean water, less corrosion was observed in the simulated tidal zone. The likely reason for this is the reduced deposition of biomass on the test fragments [27].

In terms of factors influencing the corrosion phenomenon, not only material composition and structure, but also the effects of moisture and H_2_S, reaction, temperature and microorganisms should be singled out.

The medium with which the material is in contact has a significant impact on the rate of corrosion. Aggressive media include domestic sewage which contains chlorides, sulphates, ammonium ions or aggressive carbon dioxide [28]. The biological decomposition of sulfur-containing organic compounds contributes to the production of sulfides. In addition, the anaerobic conditions that prevail in sewage systems are conducive to the formation of hydrogen sulfide, the release of which increases as the pH of the sewage decreases [29]. In the wastewater, despite the fact that the reaction was above 7, sulphates periodically increased above 250 mg/dm^3^ and chlorides above >500 mg/dm^3^. The results of other researchers’ experiments confirm a much more aggressive biogenic effect of sulfide on 304 steel than that of its inorganic counterpart [30]. Despite many proposed solutions for eliminating hydrogen sulfide [7], it remains a major problem in technical facilities. In order to reduce the environmental impact of bioaerosols and odorants or to reduce odor nuisance, facilities are most often covered, while ventilation equipped with special filters is installed. Unfortunately, the widespread encapsulation of treatment plant facilities carries the risk of hypoxia in rooms due to a low ventilation capacity, which promotes intensive production of hydrogen sulfide. Often, excessive concentrations are observed during the summer months and during rainy weather. The presence of hydrogen sulfide and high humidity promote faster oxidation of metals, leading to corrosion. Guo et al. [31] confirmed that natural changes in humidity can increase pitting corrosion, especially for small pits, while at the same time reducing large pits. Also, Cheng et al. [32] confirmed the effect of humidity changes (i.e., the occurrence of alternating wetting cycles) in terms of increasing pitting corrosion of 304 steel. The presence of H_2_S in the environment causes a denobling effect and an increase in the passive current density, in addition to significantly affecting cracking time and causing pitting corrosion [27,33]. In an acidic environment, H_2_S accelerates anodic dissolution in pits and cracks [34].

A decreasing reaction and increasing temperature amplify the release of hydrogen sulfide. This is due to the fact that *Acidithiobacillus* spp., which oxidize sulfur and produce sulfuric acid, require a pH from 2 to 5 for optimal growth and H_2_S conversion. On the other hand, research by Nielsen et al. [35] indicates that lowering the temperature of wastewater can inhibit hydrogen sulfide formation. Increased sulfide production occurs when oxygen and nitrate are extremely low, most often in pressure pipes or during sludge processing. In gravity sewers, sulfide production occurs mainly in the biological membrane (biofilm) or in a sludge. The biological decomposition of organic compounds containing sulfur leads to sulfide production and results from the activity of mainly sulfate-reducing bacteria (SRB). SRB belong to four phylogenetically distinct groups of the Bacteria and Archea domains, with the majority belonging to Deltaproteobacteria including sulfate-reducing bacteria (*Desulfovibrio*, *Desulfobacter*, *Desulfococcus*, *Desulfonema* and sulfur-reducing bacteria (e.g., *Desulfuromonas* spp.)) [2]. When conditions are changing from anaerobic to anoxic, bacteria begin to decompose nitrates instead of sulfates, as they obtain more energy from decomposing these compounds. Relevant studies confirm that describing the corrosion mechanism is very difficult, especially in wastewater from operating facilities [36].

However, species-level analysis is an appropriate approach to microbial assessment, as bacteria identification at this phylogenetic level sometimes requires very labor-intensive analyses. Identification of specific classes of bacteria on the surface of steel samples can indicate the presence of specific groups responsible for creating conditions favorable for the corrosion process. According to McIlroy et al. [24], wastewater is dominated by Proteobacteria that are mainly classified into four classes (α- β- γ- δ-Proteobacteria). Betaproteobacteria, most of which are aerobic bacteria, are of biotechnological importance and usually dominate the activated sludge due to their biodegradation properties [37]. The results of this research showed that these bacteria constitute the lowest class in terms of abundance. This may indicate a reduced oxygen concentration in the wastewater. According to research at Skagen WWTP, Betaproteobacteria constituted the most numerous group (38%), while Gammaproteobacteria and Alphaproteobacteria contents were 9% and 7 ± 2%, respectively. Deltaproteobacteria accounted for only 2 ± 1% [25]. Increased amounts of the Deltaproteobacteria class in wastewater may be indicative of a medium rich in bacteria that promote the formation of hydrogen sulfide. The Deltaproteobacteria class consists of strictly anaerobic bacteria. They constitute a heterogeneous group of bacteria that play a key role in the sulfur cycle [38]. Analysis of the biofilm from the anaerobic reactor showed that Deltaproteobacteria were the dominant class, while Alphaproteobacteria and Betaproteobacteria accounted for 22.1 and 27.8%, respectively [39].

While the steel samples that were submerged or half-submerged did not show corrosion, the steel samples suspended above the surface of the wastewater were already characterized by significant corrosion changes. The progress of corrosion is determined by the availability of oxygen. Bacteria can produce H_2_S, but there is no corrosive force on the surface. Only H_2_S with oxygen in the air forms an acid that affects the progress of corrosion. Therefore, it can be concluded that the contribution of bacteria to this process is indirect and indicates that on facilities that are vulnerable to corrosion, in addition to taking care of proper ventilation, it is advisable to regularly remove sludge deposited on the surfaces of equipment in contact with wastewater. The presence of these bacteria in the sludge can promote the growth of anaerobic bacteria that produce hydrogen sulfide; therefore, controlling the groups of bacteria responsible for this process seems crucial.

The contribution of bacteria to the corrosion process is indirect and indicates that on facilities vulnerable to corrosion, in addition to taking care of proper ventilation, it is advisable to regularly remove sludge deposited on the surfaces of equipment in contact with wastewater. The presence of these bacteria in the sludge can contribute to the excessive growth of anaerobic bacteria which produce hydrogen sulfide.

## 5. Conclusions

In the case of nickel–chromium 304 steel, its “stainlessness” notwithstanding, it has become necessary to control its chemical composition, the development of the surface grade and, above all, its phase composition.

- According to this study, delta ferrite can have a negative effect on the properties of stainless steel placed above the surface of the wastewater (A-series) and can affect pitting corrosion.
- The results showed that in a humid environment containing H_2_S, this can lead to the intensification of corrosion from the center of corrosion, propagating deep into the pits after the damage of the passive layer under favorable conditions of temperature, humidity, insufficiently efficient ventilation and the impact of microorganisms.
- The proposed approach to microbiological assessment by determining the classes of bacteria can be a useful tool for evaluating wastewater for aggressiveness. The early identification of bacteria can also reduce financial losses of technical facilities.
- As a result of the FISH analysis, the δ-proteobacteria class dominated in the sludge taken from the steel samples submerged in wastewater. This confirms the presence of bacteria involved in the reduction of sulfur or sulfate compounds, resulting in the formation of hydrogen sulfide, which is the main cause of increased corrosion of steel plates hung above the surface of the wastewater.

## Figures and Tables

**Table 1:** Chemical composition of the stainless steel samples.

Fe	C	Ni	Cr	Mn	Si	N	P	S
[wt.%]								
Bal.	0.037	8.04	18.13	1.28	0.42	0.057	0.029	0.002

**Table 2:** Oligonucleotide probes and examples of bacteria present in wastewater [http://probebase.csb.univie.ac.at (accessed on 15 September 2023)].

Probe	Sequence	Formamide [%]	Fluorescent Dye	Examples of Mechanism and Bacteria Categories from Each Class
ALF968 ^1^	5′-GGT AAG GTT CTG CGC GTT-3′	20	Green	Alphaproteobacteria
				nitrite-oxidizing bacteria *Nitrobacter* *denitrifying (nitrate-reducing) bacteria* *Paracoccus denitrificans*
BET42a	5′-GCC TTC CCA CTT CGT TT-3′ ^[a]^	35	Red	Betaproteobacteria
				ammonia-oxidizing bacteria *Nitrosomonas* iron-oxidizing bacteria *Gallionella ferruginea*
Gam42a	5′-GCC TTC CCA CTT CGT TT-3′ ^[a]^	35	Red	Gammaproteobacteria
				S-oxidizing (sulfur-oxidizing) bacteria *Acidithiobacillus thiooxidans*
DELTA495a ^2^	5′-AGT TAG CCG GTG CTT CCT-3′ ^[a]^	35	Green	Deltaproteobacteria
				sulfur-reducing bacteria *Desulfuromonas* iron- and manganese—reducing bacteria sulfate-reducing microbes *Desulfovibrio aestuarii*

^1^ except Rickettsiales; ^2^ most Deltaproteobacteria and most Gemmatimonadetes; ^[a]^ require the use of a competitor.

## Data Availability

Not applicable.
